# Identification of mitophagy-related genes with potential clinical utility in myocardial infarction at transcriptional level

**DOI:** 10.3389/fcvm.2023.1166324

**Published:** 2023-05-26

**Authors:** Zhikai Yang, Liang Sun, Hua Wang

**Affiliations:** ^1^ Department of Cardiology, Beijing Hospital, National Center of Gerontology; Institute of Geriatric Medicine, Chinese Academy of Medical Sciences, Beijing, China; ^2^ The NHC Key Laboratory of Geriatrics, Institute of Geriatric Medicine, Chinese Academy of Medical Sciences, Beijing Hospital/National Center of Gerontology of National Health Commission, Beijing, China

**Keywords:** machine learning—ML, mycardial infarction, mitophagy, signature gene, immune cells infiltration

## Abstract

**Background:**

Myocardial infarction (MI) ranks among the most prevalent cardiovascular diseases. Insufficient blood flow to the coronary arteries always leads to ischemic necrosis of the cardiac muscle. However, the mechanism of myocardial injury after MI remains unclear. This article aims to explore the potential common genes between mitophagy and MI and to construct a suitable prediction model.

**Methods:**

Two Gene Expression Omnibus (GEO) datasets (GSE62646 and GSE59867) were used to screen the differential expression genes in peripheral blood. SVM, RF, and LASSO algorithm were employed to find MI and mitophagy-related genes. Moreover, DT, KNN, RF, SVM and LR were conducted to build the binary models, and screened the best model to further external validation (GSE61144) and internal validation (10-fold cross validation and Bootstrap), respectively. The performance of various machine learning models was compared. In addition, immune cell infiltration correlation analysis was conducted with MCP-Counter and CIBERSORT.

**Results:**

We finally identified ATG5, TOMM20, MFN2 transcriptionally differed between MI and stable coronary artery diseases. Both internal and external validation supported that these three genes could accurately predict MI withAUC = 0.914 and 0.930 by logistic regression, respectively. Additionally, functional analysis suggested that monocytes and neutrophils might be involved in mitochondrial autophagy after myocardial infarction.

**Conclusion:**

The data showed that the transcritional levels of ATG5, TOMM20 and MFN2 in patients with MI were significantly different from the control group, which might be helpful to further accurately diagnose diseases and have potential application value in clinical practice.

## 1. Introduction

In recent years, the global average life expectancy has been on the rise due to improved quality of life. However, this has led to a surge in the number of individuals suffering from cardiovascular diseases, which claim the lives of 3.8 million men and 3.4 million women worldwide each year, according to the World Health Organization (1). Among these diseases, myocardial infarction (MI) is the most severe type of cardiovascular disease and the primary reason for the yearly increase in coronary artery disease mortality (2). Acute myocardial infarction (AMI), which encompasses various pathological changes such as ischemia and necrosis, represents the early stage of MI (3). Currently, the diagnosis of MI is typically reliant on electrocardiogram, physical examination, and certain biomarkers (4). MI is frequently encountered during clinical and forensic autopsies, and their diagnosis can present a challenge, particularly when there is no evidence of acute coronary occlusion (5). Hence, there is a need to investigate novel biomarkers to achieve a more precise diagnosis of the disease and gain a fresh understanding of it.

Presently, thrombolysis or percutaneous coronary intervention (PCI) are effective treatment methods for coronary blood revascularization after myocardial infarction. However, these treatments may induce cardiac ischemia/reperfusion (I/R) injury and other pathologies, including reactive oxygen species (ROS) production, Ca^2+^ overload, and mitophagy dysregulation (6–8). Mitochondria are the primary energy factories in heart muscle cells and play a vital role in maintaining cardiac structure and function. When a myocardial infarction occurs, damaged mitochondria may be produced, which promotes oxidative stress and apoptosis. Mitophagy, therefore, is essential in maintaining cardiac structure and function (9–11). Studies indicate that irisin-activated optic atrophy 1 (OPA1) could increase PINK1/Parkin-mediated mitophagy, prevent myocardial cell damage after myocardial infarction, and reverse cardiac dysfunction caused by ischemia, playing a pivotal role in maintaining myocardial cell vitality and mitochondrial function following myocardial infarction (12). Mitophagy is a type of selective autophagy that removes mitochondria with abnormal functions in cells. In related studies, Beclin1^+/−^ and FUN14 domain-containing 1 (FUNDC1) knockout and transgenic mouse models, in conjunction with starvation and myocardial infarction models, suggest that mitophagy, rather than general autophagy, plays a cardioprotective role by regulating mitochondrial function (13). Another research team discovered that the level of mitophagy decreased significantly after myocardial infarction in a mouse myocardial infarction model. Moreover, the expression of nuclear dot protein 52 (NDP52) that promotes mitophagy could reduce myocardial damage caused by myocardial infarction. This suggests that myocardial damage caused by myocardial infarction may be closely related to decreased autophagy. Therefore, activation of autophagy may have a protective effect on myocardial cells (6).

The mechanism of myocardial injury in patients with myocardial infarction remains to be fully elucidated and identifying intervention targets may have significant clinical value. As such, exploring the relationship between myocardial infarction and mitophagy is imperative to develop additional therapeutic approaches and improve myocardial infarction prognosis. In this article, we used a diverse range of bioinformatics and ML methods to identify signature genes for myocardial infarction and mitophagy.

## 2. Methods and materials

### 2.1. Data sources and preparation

All data are free from Gene Expression Omnibus (GEO). The GSE59867, GSE62646, GSE61144 datasets were available in NCBI (https://www.ncbi.nlm.nih.gov/geo/). And these datasets were based on GPL6106 platform of Sentrix Human-6 v2 Expression BeadChip and GPL6244 platform of [HuGene-1_0-st] Affymetrix Human Gene 1.0 ST Array [transcript (gene) version] (14–16). GSE59867 and GSE62646 were combined as a training set and GSE61144 as a test set for external validation. All blood samples taken were within 24 h of the occurrence of myocardial infarction. Finally, the training set had 139 MI (Myocardial Infarction) and 60 CT (Stable coronary artery disease), and the testing set had 14 MI and 10 CT. The genes of mitophagy were downloaded from REACTOME (https://reactome.org). PCA (Principal Component Analysis) plots were also produced to show the differences before and after data merging (Supplementary Figure S1A, B).

### 2.2. Differences in the expression of all genes

Using the limma package of R software (17), all genes (*n* = 18,837) between myocardial infarction group and stable coronary disease group were explored, then it intersects with the mitophagy genes. This differential gene expression was visualized using a volcano plot (only those with *p* < 0.05 were labeled), while the comparison between MI group and control group was shown utilizing a heatmap.

### 2.3. Pick up signature gene

The key genes were obtained by intersection of all genes and mitophagy gene set. ML algorithm is more suitable than traditional statistical methods when dealing with large, complex and high latitude data (18). Thus, we explored genes using three machine learning (ML) algorithms, namely Support Vector Machine-Recursive Feature Elimination (SVM-RFE), least absolute shrinkage and selection operator (LASSO) as well as random forest.

The SVM-RFE algorithm of package “caret” is one of the most popular gene selection methods at present, which is designed for binary classification problems (19). The penalty parameter tuning was performed using the LASSO algorithm of the package “glmnet”, after tuning the penalty parameters in a 10-fold cross-validation procedure. This method is more effective than regression analysis in assessing high-dimensional data (20). The genes were further classified using the R package “randomforest”. An average error rate of key genes determines how many variables to include in a random forest model (21). We then calculated error rates for 1–1,000 trees. The top 10 genes were selected by SVM model and random forest model respectively. The genes with the highest accuracy were selected in lasso model. Signature genes of myocardial infarction were identified by the intersection of the three ML algorithms. The AUC was used to evaluate the models' diagnostic ability. The AUC greater than 0.7 illustrate good diagnostic effect.

### 2.4. Building machine learning models

Finally, it is decided to construct different supervised machine learning models with three genes as variables, which are decision tree (DT), K-nearest neighbor (KNN), random forest (RF), support vector machine (SVM) and logistic regression (LR). Their accuracy, precision, recall, F1 score, and AUC were compared, and the final value for each parameter was obtained by taking the average of the values calculated through 5-fold cross-validation.

### 2.5. Internal validation and external validation

After the model performance comparison, a logistic regression model was constructed, and draw 1,000 bootstrap samples, and the model was rebuilt each time to draw the ROC. Then the performance of the model was evaluated by 10-fold cross-validation of 199 samples. About external validation, the GSE61144 dataset was downloaded (16), and 24 samples were predicted to verify the generalization ability of the model.

### 2.6. Immune cell infiltration

The package “IOBR” was used to analysis (22). CIBERSORT is a method that deconvolutes human immune cell subtype expression matrices using linear support vector regression to investigate the correlation between these genes and immune cells (23). Additionally, the microenvironment cell population (MCP)-counter algorithm was utilized. This method can reliably quantify the abundance of various immune based on transcriptomic data for each sample (24). The correlation between some genes and some immune cells was analyzed by spearman method.

### 2.7. Statistical analysis

**Figure 1:**
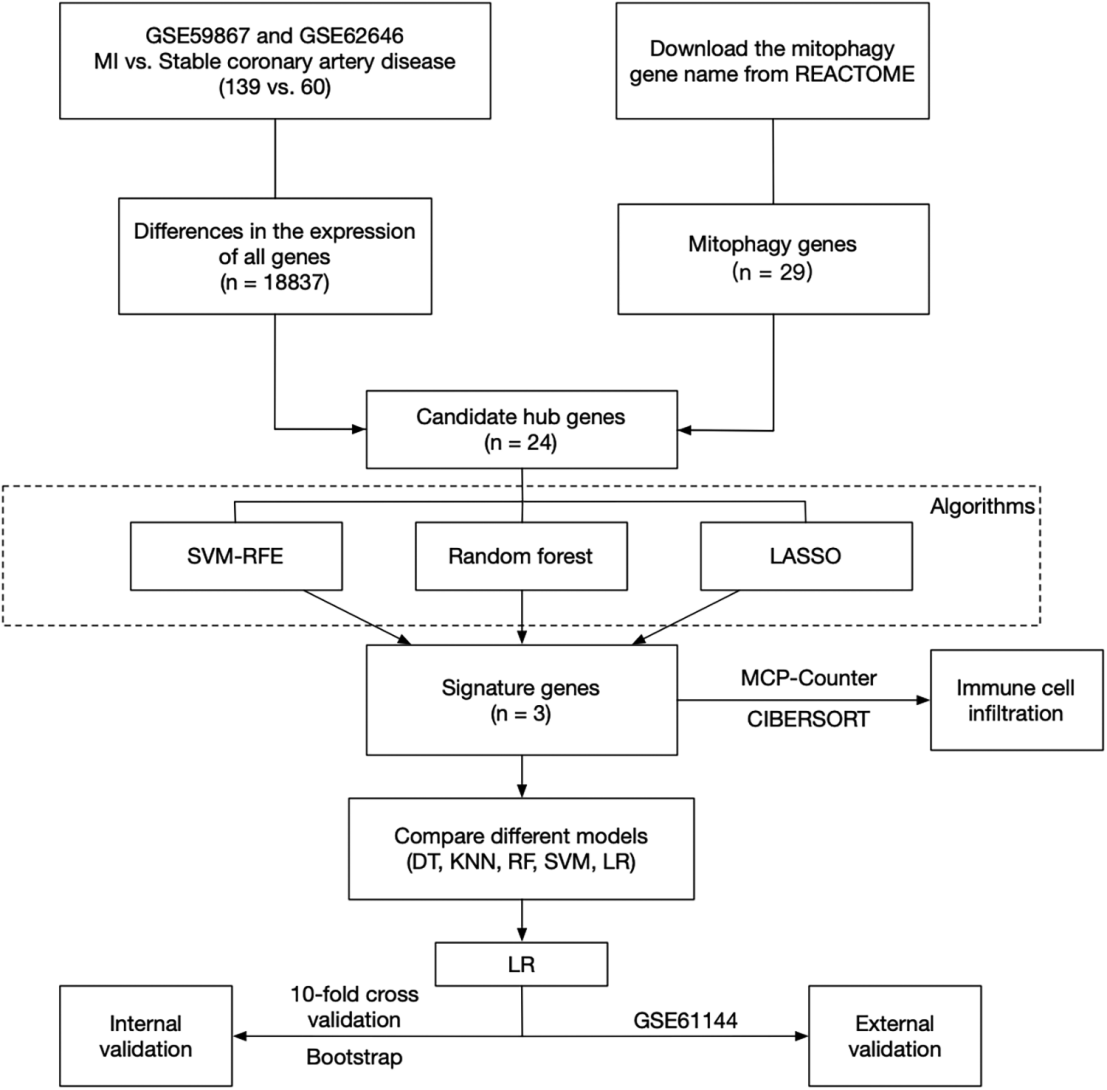
The flow chart of this research.

R (version 4.2.2) was used for all statistical analyses in this study. In all cases, a default *p* or adjust. *p* of less than 0.05 was considered statistically significant. All *p* values were two-sided tests. According to Figure 1, the flow chart about this research was as follows.

## 3. Results

### 3.1. Identification of differentially expressed mitophagy genes

**Figure 2:**
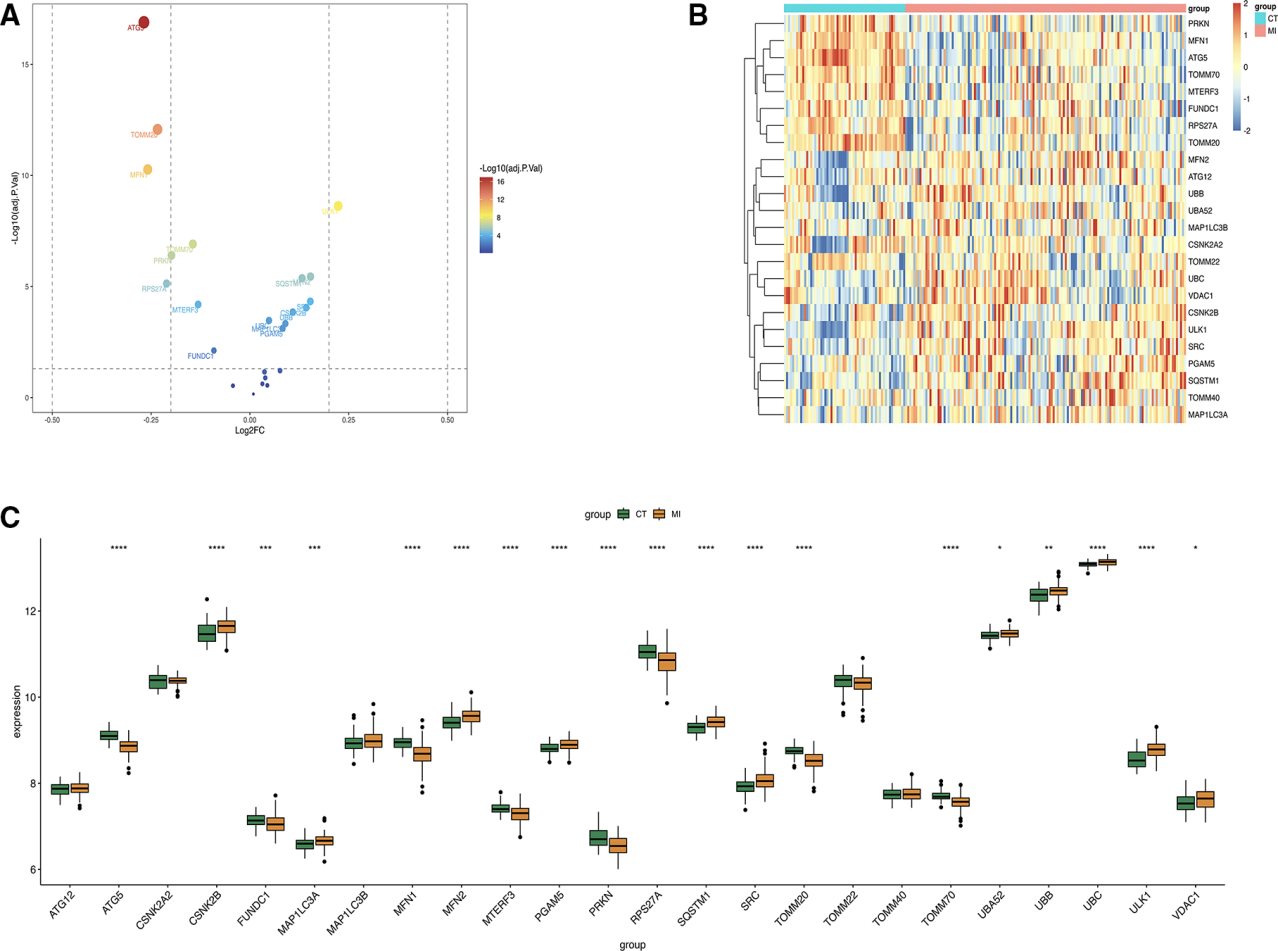
(**A**) Volcano plot about mitophagy genes. (**B**) Heat map of the 24 differentially expressed mitophagy genes in MI samples and healthy samples. (**C**) Boxplot of 24 gene expression levels.

The intersection of all genes derived from “limma” package and mitophagy genes was taken, and 24 related genes were obtained. Only 17 of them were statistically different. The volcano plot was made to display 24 genes of mitophagy (Figure 2A), the heatmap showed the genes between MI group and control group (Figure 2B) and made the boxplot (Figure 2C).

**Figure 3:**
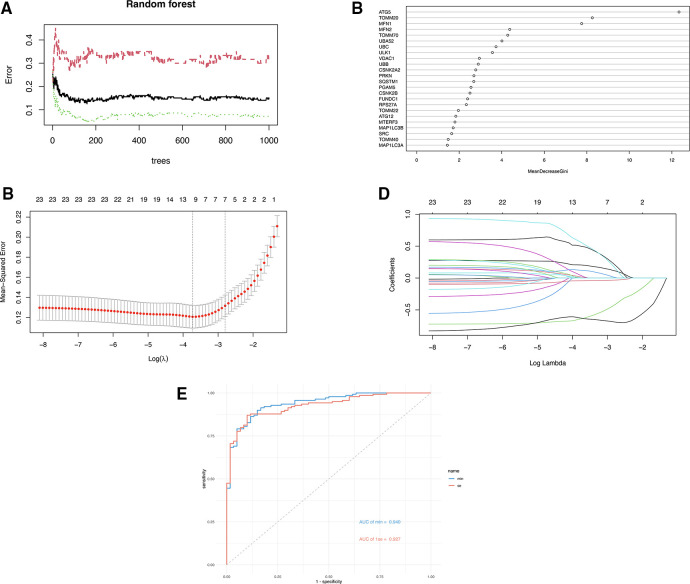
(**A**) The error rate confidence intervals for random forest model. (**B**) The relative importance of genes in random forest model. (**C**) Penalty plot of the LASSO model with error bars denoting standard errors. (**D**) The least absolute shrinkage and selection operator (LASSO) coefficient profiles. (**E**) ROC curves of two gene sets based on LASSO algorithm.

**Figure 4:**
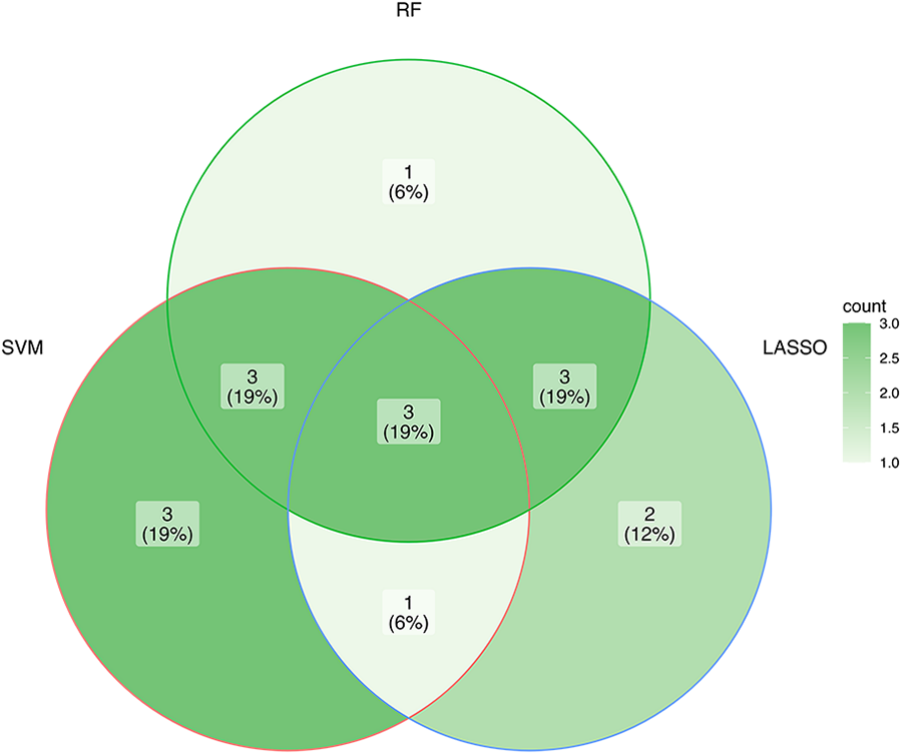
The interaction of the three algorithms.

Three ML algorithms were utilized to pick up the signature genes from 24 genes. For the SVM-RFE and random forest algorithms select top 10 of genes respectively (Figures 3A, B). The smallest error is on 159 trees. And LASSO analysis selected the genes with the highest accuracy (based on ROC) (Figures 3C–E). Table 1 presents the genes under consideration. Finally, the three genes were determined by taking the intersection of the three algorithms, including ATG5, TOMM20, MFN2 (Figure 4). Detailed information about the genes is shown in Table 2.

**Table 1:** The key genes from different algorithms.

SVM-RFE	Random forest	LASSO
TOMM70, ATG5, MFN1, PGAM5, MAP1LC3A, ATG12, TOMM20, RPS27A, ULK1, SQSTM1	ATG5, TOMM20, MFN1, TOMM70, UBA52, SQSTM1, UBC, MFN2, ULK1, CSNK2A2	Min: MFN2, TOMM20, PGAM5, UBC, MAP1LC3B, UBA52, SQSTM1, ATG5, PRKN 1se: MFN2, TOMM20, UBC, UBA52, SQSTM, ATG5, PRKN

**Table 2:** Detailed information on three genes.

Official full name	Official symbol	Gene ID	Location	Function
Autophagy related 5	ATG5	9474	6q21	The protein encoded by this gene, in combination with autophagy protein 12, functions as an E1-like activating enzyme in a ubiquitin-like conjugating system. The encoded protein is involved in several cellular processes, including autophagic vesicle formation, mitochondrial quality control after oxidative damage, negative regulation of the innate antiviral immune response, lymphocyte development and proliferation, MHC II antigen presentation, adipocyte differentiation, and apoptosis.
Translocase of outer mitochondrial membrane 20	TOMM20	9804	1q42.3	Enables protein-transporting ATPase activity and unfolded protein binding activity. Involved in protein targeting to mitochondrion. Located in mitochondria-associated endoplasmic reticulum membrane and mitochondrial outer membrane.
Mitofusin 2	MFN2	9927	1p36.22	This gene encodes a mitochondrial membrane protein that participates in mitochondrial fusion and contributes to the maintenance and operation of the mitochondrial network. This protein is involved in the regulation of vascular smooth muscle cell proliferation, and it may play a role in the pathophysiology of obesity.

### 3.2. Diagnostic efficacy in predicting MI

**Figure 5:**
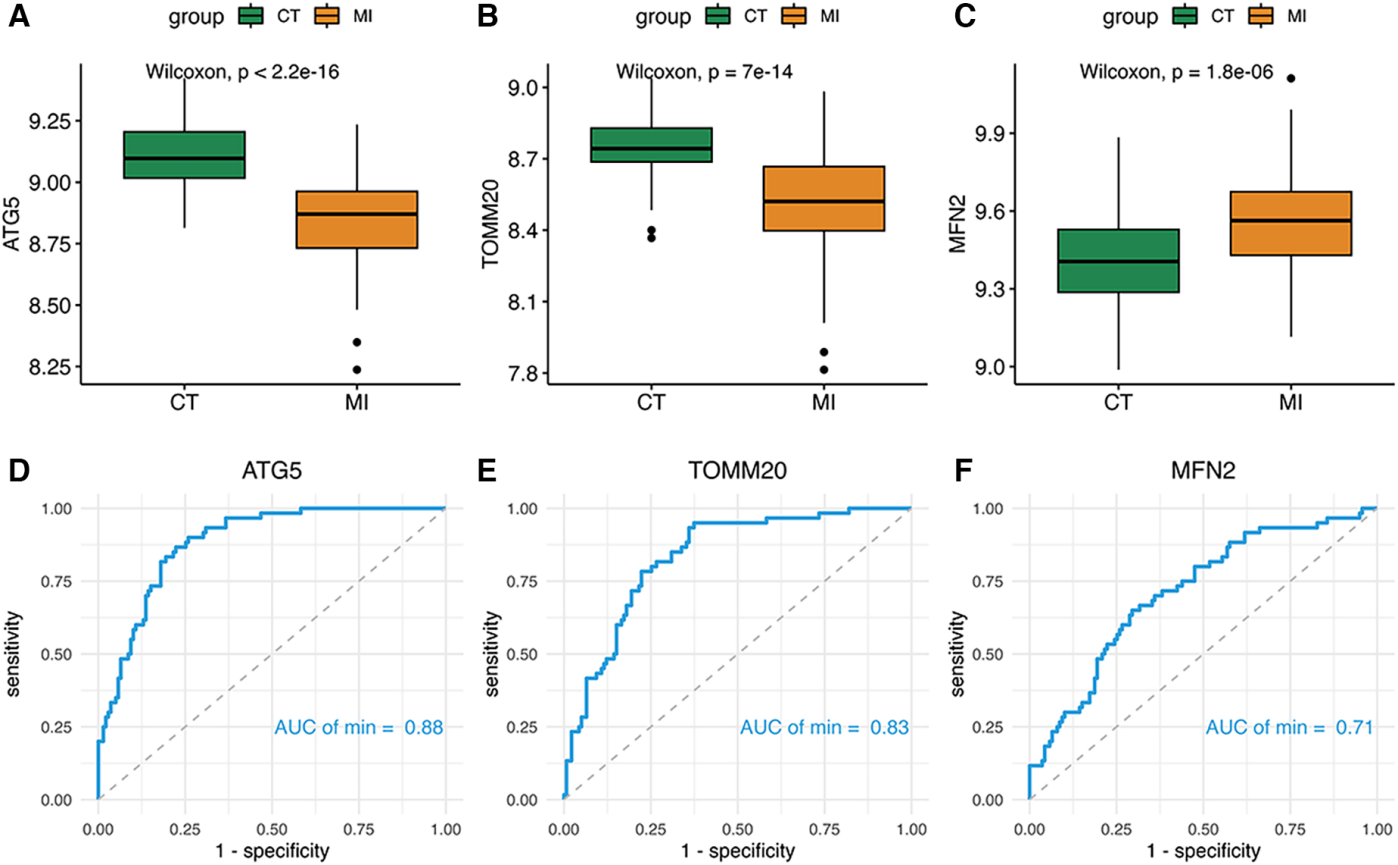
(**A–F**) Boxplot and ROC of three diagnostic genes.

Two of the signature genes were highly expressed in the MI group and one was underexpressed, suggesting that three genes could have a latent ability to diagnosis MI (Figures 5A–C). Moreover, the AUC of the ROC of these key genes was 0.88 of ATG5, 0.83 of TOMM20, 0.71 of MFN2 respectively (Figures 5D–F).

### 3.3. Model comparison

The metrics of each model are shown in Table 3. DT, KNN and SVM have the highest accuracy of 0.814. LR has the highest AUC (0.915) and precision (0.739). DT has the highest F1-score (0.702). All algorithms have recall rates of more than 50%. The roc curve of each model is shown in the attachment (Supplementary Figure S3), along with the AUC shown in boxplot (Supplementary Figure S4).

**Table 3:** Performance of supervised machine learning algorithms in MI prediction.

Algorithms	Accuracy	Precision	Recall	F1-score	AUC
DT	**0** **.** **814**	0.692	0.712	**0**.**702**	0.816
KNN	**0**.**814**	0.683	**0**.**719**	0.701	0.881
RF	0.799	0.679	0.641	0.659	0.905
SVM	**0**.**814**	0.736	0.641	0.685	0.897
LR	0.808	**0**.**739**	0.618	0.673	**0**.**915**

Bold values denote the highest value in the column.

**Figure 6:**
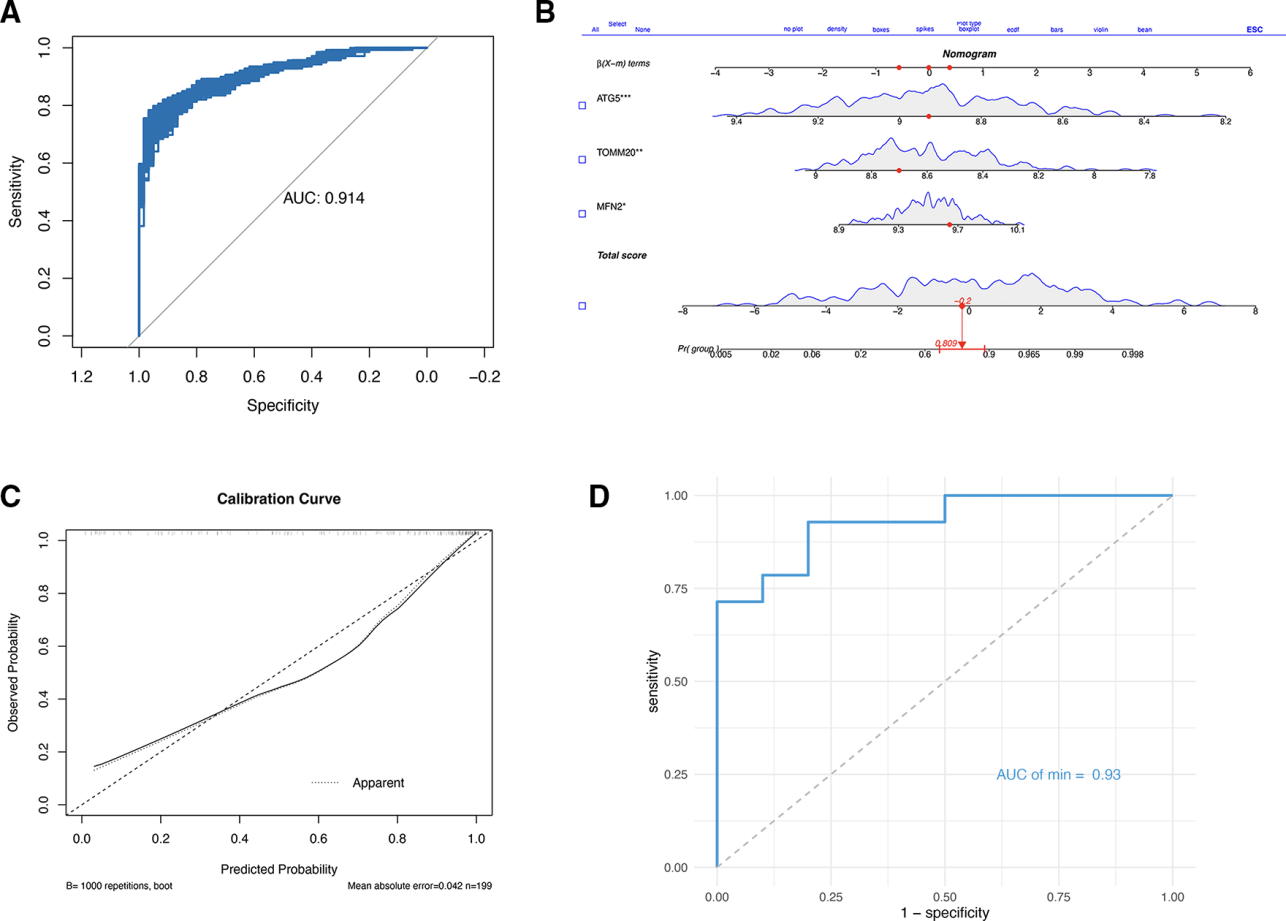
(**A**) Logical regression and bootstrap validation based on three gene constructs. (**B**) Nomogram to predict the occurrence of MI. (**C**) Calibration curve to assess the predictive power of the logistic model. (**D**) ROC based on GSE61144.

Subsequently, a multi-factor logistic regression model was constructed with these three genes, the AUC of the model was obtained by using 10-fold cross-validation, and then the average value was obtained (Supplementary Table S1) (0.915), then the fitting effect of the model was verified with the training set, and 1,000 bootstrap samples were drawn (Figure 6A). A nomogram was generated using the logistic regression model (Figure 6B). A calibration curve was drawn to evaluate the classifier's predictive ability, which revealed minimal differences between the predicted and actual MI risks. This result indicates the model's high effectiveness, as depicted in Figure 6C. Finally, the data set GSE61144 was used for external verification to obtain the ROC (Figure 6D), the AUC was 0.93. These data indicate that three signature genes have a good ability to predict MI. The results of bootstrap method were displayed in Supplementary Figure S2.

### 3.4. Immune cell infiltration

**Figure 7:**
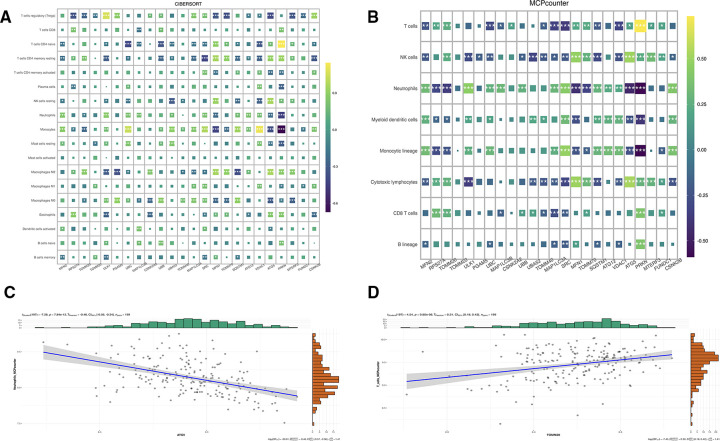
(**A**) Correlation map of genes and immune cells based on CIBERSORT algorithm. (**B**) Correlation map of genes and immune cells based on MCPcounter algorithm. (**C**) Correlation between ATG5 and neutrophils based on MCPcounter calculations. (**D**) Correlation between TOMM20 and T cells based on MCPcounter calculations.

**Figure 8:**
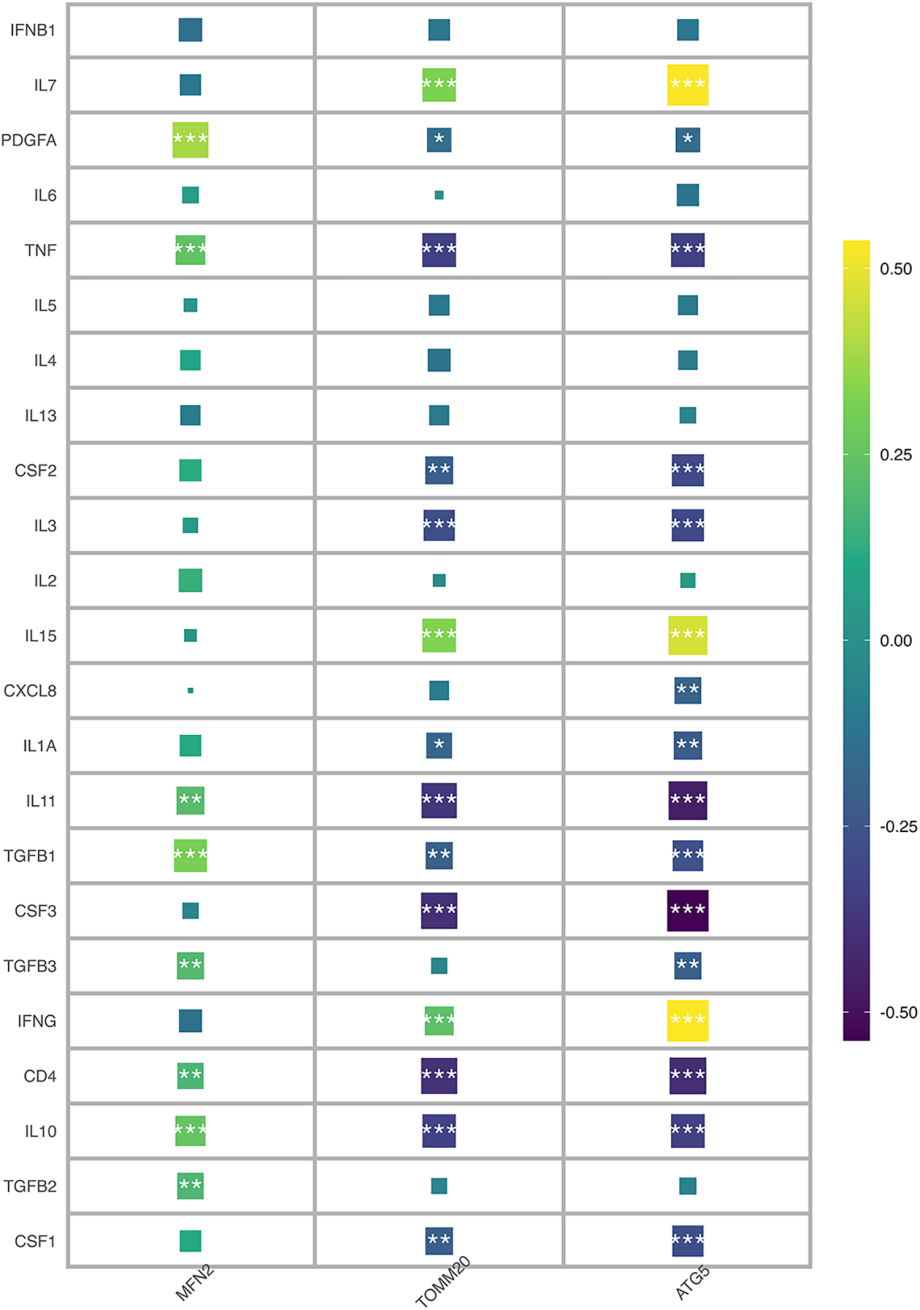
Correlation diagram of three diagnostic genes and inflammatory factors.

Immune characteristics were evaluated by CIBERSORT method, 24 genes and T cells CD4 naive, neutrophils, monocytes, macrophages M2, macrophages M0 has a strong relevance among which MFN2 was positively correlated with monocytes and neutrophils, and negatively correlated with T cell CD4 naive and T cell CD4 memory resting, and ATG5 was positively correlated with T cell CD4 memory resting and NK cells resting, and negatively correlated with Tregs and monocytes (Figure 7A). According to MCP-counter method, 24 genes were strongly correlated with T cells, NK cells, neutrophils, monocytic lineage and cytotoxic lymphocytes (Figure 7B), there was a negative correlation between ATG5 and neutrophils (Figure 7C). And a positive correlation between TOMM20 and T cells (Figure 7D). Subsequently, correlation diagrams of 3 diagnostic genes and inflammatory factors were also made, indicating that the 3 genes screened in this study were strongly correlated with TNF, IL11, TGFB1, CD4 and IL10 (Figure 8).

## 4. Discussion

Globally, CVDs continue to be the primary cause of mortality on a global scale, and they also play a significant role in the global burden of illness (25). MI is also the leading cause of death and morbidity (2). Employing bioinformatics techniques, this study identified differences in genes in patients with myocardial infarction and patients in stable condition. Thereafter, the SVM-RFE algorithm, random forest algorithm, and LASSO algorithm were utilized to identify mitophagy genes associated with myocardial infarction, including ATG5, TOMM20, and MFN2. Next, various supervised machine learning algorithm models were constructed, with logistic regression exhibiting the highest AUC value (0.915). Subsequently, a logistic regression model was constructed to validate the genes internally and externally. Finally, the CIBERSORT algorithm and MCP-counter algorithm were employed to explore the correlation between immune cell infiltration and the signature genes.

Cardiac mitochondria are responsible for oxidative phosphorylation (OxPhos) to produce ATP energy, which is crucial for cardiac function (26). Therefore, mitochondrial dysfunction may represent a latent mechanism in the pathogenesis or prognosis of myocardial infarction. Mitophagy is known to increase during cardiac stress and injury, helping to clear damaged mitochondria and prevent oxidative damage as well as cell death (27, 28). Therefore, additional research is necessary to ascertain whether the induction of mitophagy following myocardial infarction is indicative of a distinct prognosis.

ATG5 is a pivotal component of the ATG12-ATG5-ATG16 complex that plays a crucial role in promoting phagophoric membrane elongation in autophagic vesicles (29, 30). Wang et al. have demonstrated that Atg5-dependent autophagy is an evolutionarily conserved autodigestive process that is essential for induced cardiomyocyte reprogramming (iCM) (31). Meanwhile, Schriner et al. suggest that increased autophagy activity may lead to thinner and longer survival time of Atg5 transgenic mice (29). Further studies have revealed that ATG5 defects can lead to increased ubiquitination levels in heart tissue, sarcomere disorders, mitochondrial aggregation, and other related abnormalities. Knockout of the ATG5 gene leads to myocardial cell necrosis and increased cross-sectional area of myocardial cells, indicating that ATG5 is involved in myocardial hypertrophy and obesity, and the regulation of lipid metabolism (32–34). Although few studies have explored the relationship between ATG5 and AMI, we speculate that ATG5 is related to MI.

Translocase of Outer Mitochondrial Membrane 20 (TOMM20). When intracellular mitochondrial autophagy occurs, mitochondrial degradation occurs, resulting in the reduction of marker proteins in the inner and outer membrane of mitochondria. It has been reported to be involved in many cancers (35, 36). Studies have demonstrated a correlation between increased TOMM20 expression and elevated mitochondrial mass in certain tumors (37). TOMM20 expression directly impacts mitochondrial processes, such as ATP production and membrane potential maintenance (37). Nonetheless, the precise manner in which TOMM20 expression contributes to tumor development and progression remains unclear. In addition, TOMM20 has also been used to evaluate the degree of mitophagy (38). The decreased expression of TOMM20 can greatly reduce the damage of myocardial mitochondria and reduce the apoptosis of myocardial cells (39). The reason for this phenomenon in this study may be that the blood samples were collected within 24 h after the occurrence of myocardial infarction, and this phenomenon occurs in order to reduce the damage of the heart.

Mitochondrial fusion associated protein 2 (Mfn2), mostly found in mitochondria, is involved in inhibiting Ras-Raf-MEK-ERK/MAPK and Ras-PI3K-Akt signaling pathways, thereby suppressing proliferation and promoting apoptosis of vascular smooth muscle cells (40). Overexpression of Mfn1 or Mfn2 has been found to inhibit the opening of the mitochondrial permeability transition pore (mPTP) and reduce cell death after myocardial ischemia-reperfusion injury (41). However, Mfn2 deficiency has been shown to impair cardiac function, cause heightened myocardial fibrosis, increase mitochondrial damage, and worsen oxidative stress (42). Furthermore, the increased expression of Mfn2 has been reported to contribute to mitochondrial hyperfusion (43).

In Figures 7A, B, it is evident that a significant proportion of genes are strongly correlated with monocytes and neutrophils. Monocytes, which rapidly accumulate in the injured area after myocardial injury, exhibit a pro-inflammatory and phagocytic role on necrotic substances, as indicated by previous studies, with the expression of cytokines and growth factors in the infarct area, the environment in the infarct area changes, and the infiltrated monocytes transform into mature macrophages (44). Furthermore, neutrophils are known to exert an anti-inflammatory effect through apoptosis-related mechanisms. Specifically, they secrete factors such as annexin A1 and lactoferrin, which inhibit further recruitment of neutrophils and promote macrophage aggregation to accelerate the apoptosis process. Neutrophils also stimulate macrophages to release anti-inflammatory substances such as IL-10 and TGF-*β* by activating the anti-inflammatory program in macrophages (45). This may explain why these factors have a significant meaning in Figure 8. Recent studies suggest that MFN2, in addition to regulating mitochondrial fusion, has the capacity to regulate immune responses (46). However, the mechanisms underlying the effect of mitophagy on myocardial infarction through immune cells warrant further exploration.

Most drugs that target mitophagy are non-specific and may affect other cells. Thus, identifying new therapeutic approaches to regulate mitophagy is crucial (47). The three genes discussed in this paper, namely ATG5, TOMM20, and MFN2, may provide potential targets for improving the prognosis of myocardial infarction. Myocardial infarction is a multifaceted condition that results from the heart's inability to adequately fulfill the metabolic needs of body tissues, ultimately leading to impaired function. Multiple research studies have demonstrated the indispensability of mitophagy as a critical protective mechanism for repairing damage associated with this syndrome.

The strength of this study lies in the use of bioinformatics methods and multiple machine learning algorithms to identify critical genes associated with myocardial infarction and mitophagy. Nonetheless, certain limitations and deficiencies must be acknowledged. Firstly, the study relied mainly on public databases, which may introduce bias due to the small amount of data. Nevertheless, our results were validated using K-fold cross-validation and bootstrap resampling, which demonstrate their reliability and generalizability to some extent. Secondly, the blood samples were collected within one day after myocardial infarction, and therefore the findings pertain to short-term effects. Furthermore, further studies involving larger clinical samples are required to confirm our results. Finally, the mechanism underlying the immunological infiltration of the selected genes remains to be elucidated.

## Conclusion

With bioinformatics analysis on a GEO dataset and utilizing three distinct machine learning algorithms, we successfully identified three mitophagy-related genes closely associated with MI. Furthermore, a prediction model with desired accuracy has been established. Our research provides critical insights into the molecular mechanisms underlying MI and mitophagy, thereby offering potential valuable avenues for further investigation.

## Data Availability

The datasets presented in this study can be found in online repositories. The names of the repository/repositories and accession number(s) can be found in the article/supplementary material. Further queries can be sent to the corresponding author(s).
