# Supplementary material for: Identification of mitophagy-related genes with potential clinical utility in myocardial infarction at transcriptional level
Source: Front Cardiovasc Med. 2023 May 26;10:1166324. doi: 10.3389/fcvm.2023.1166324 (PMC10250750; doi:10.3389/fcvm.2023.1166324)
Supplement: Supplementary file 1 [file Table4.docx]

Suppl.Image

Figure S1A- Principal component analysis of pre-merger data


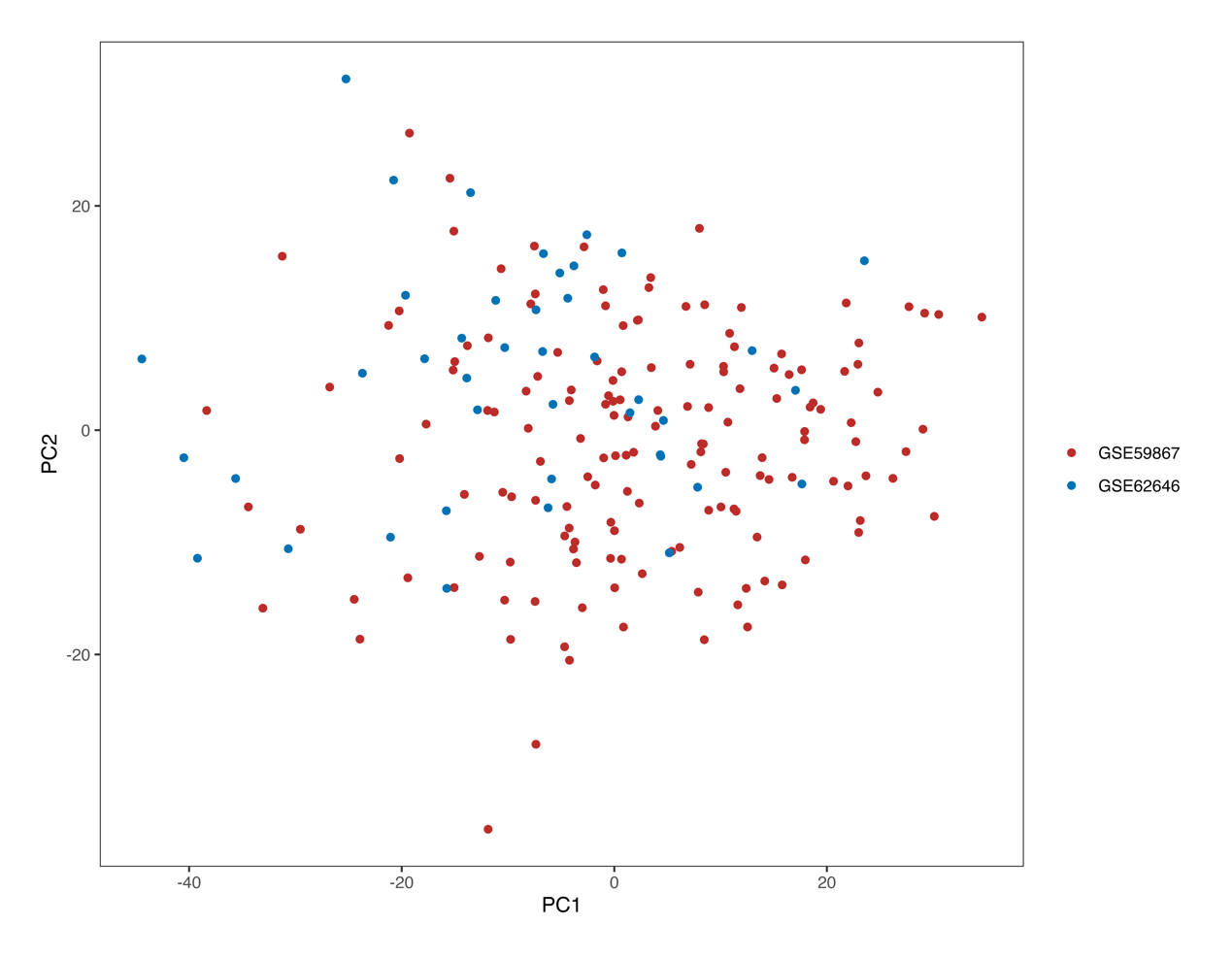


Figure S1B- Principal component analysis for the combined data


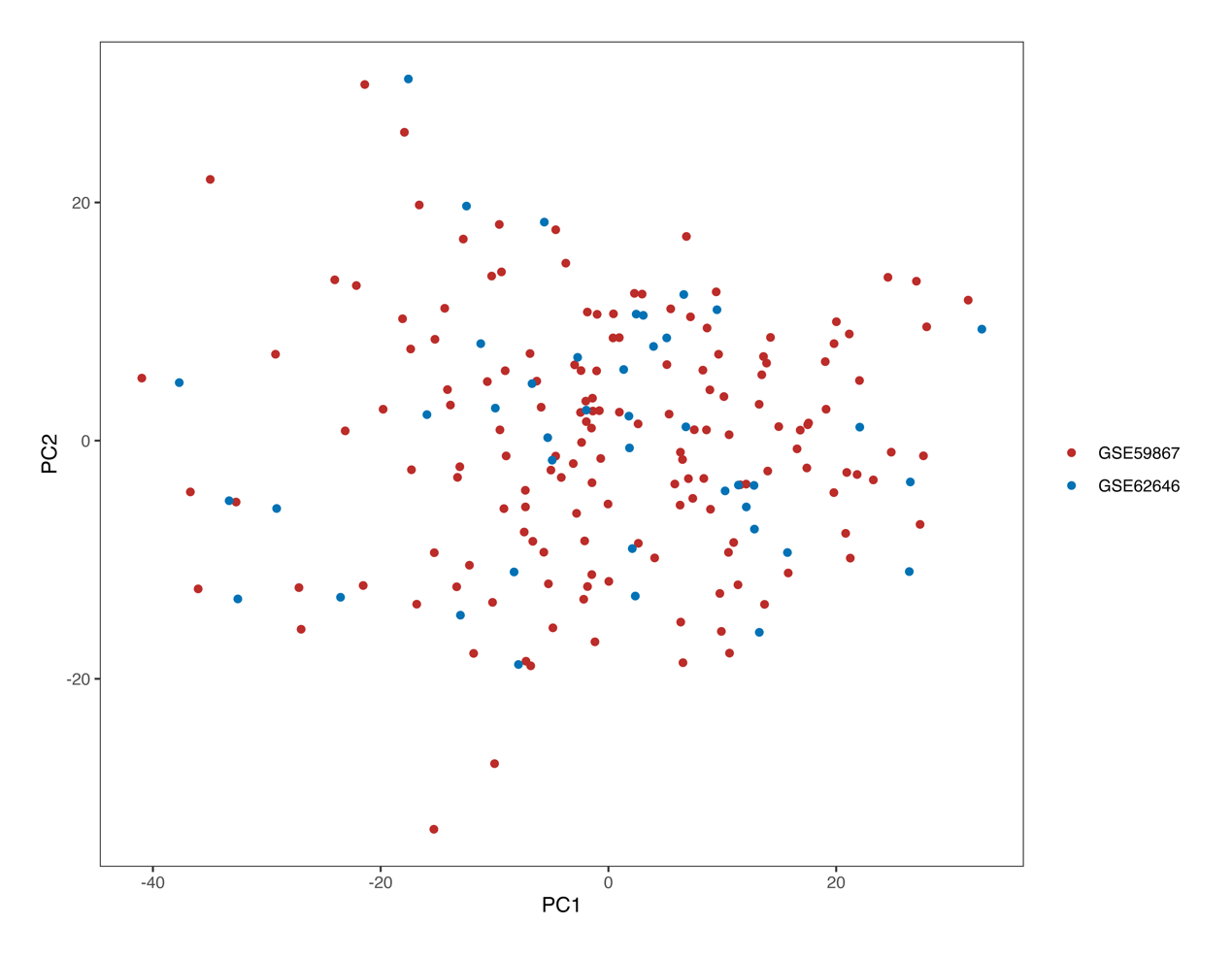


Figure S2- The bootstrap of logistic regression model

Figure S3- The ROC of models


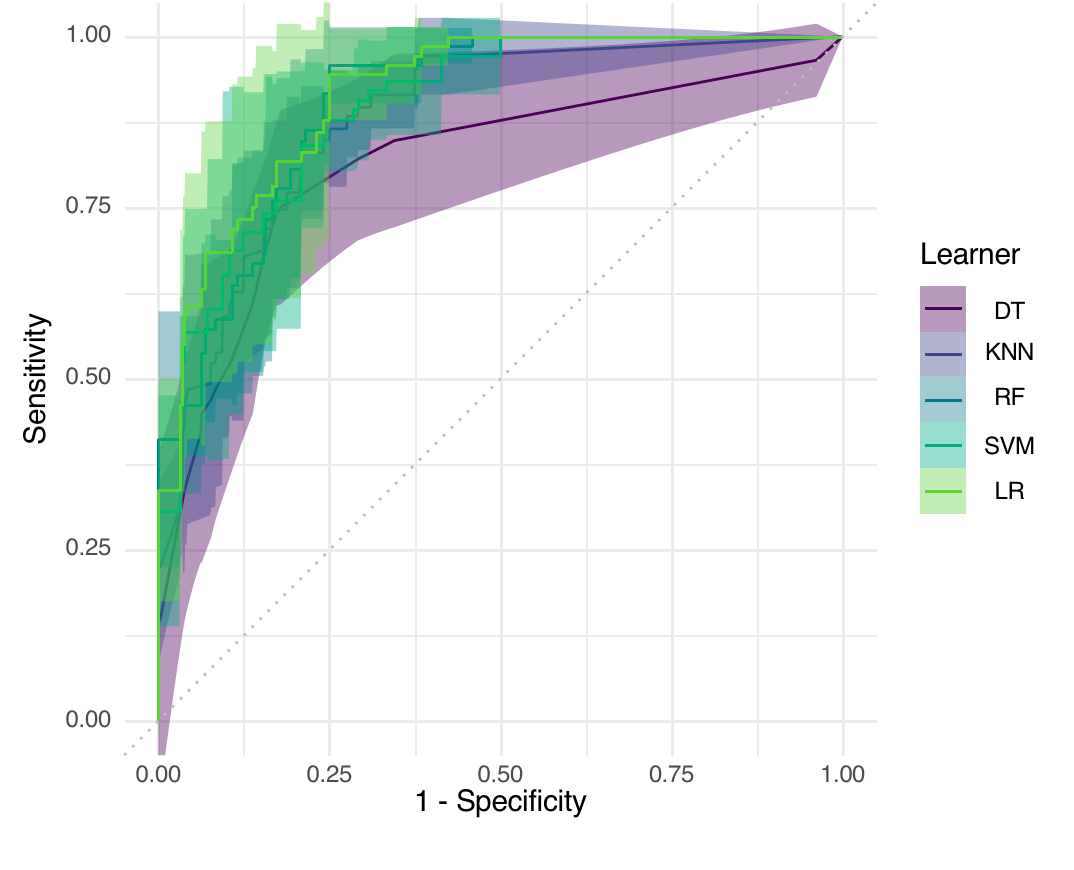


Figure S4- The AUC of different models


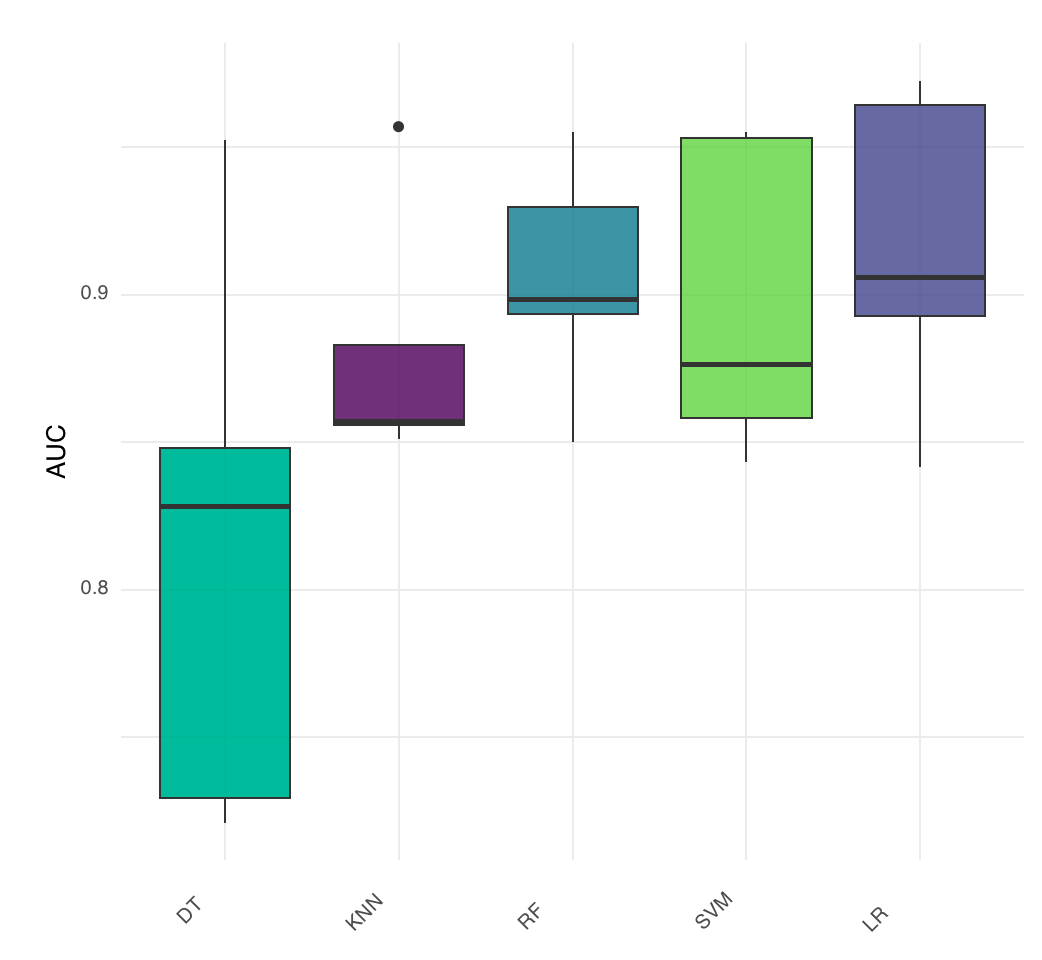


Table S1 10 fold cross-validation of logistic regression model

| Iteration | AUC |
| --- | --- |
| 1 | 0.844 |
| 2 | 0.964 |
| 3 | 0.920 |
| 4 | 0.845 |
| 5 | 0.967 |
| 6 | 0.934 |
| 7 | 0.952 |
| 8 | 0.934 |
| 9 | 0.972 |
| 10 | 0.814 |
| Average | 0.915 |
